# An integrated research framework combining genomics, systems biology, physiology, modelling and breeding for legume improvement in response to elevated CO_2_ under climate change scenario

**DOI:** 10.1016/j.cpb.2020.100149

**Published:** 2020-06

**Authors:** Paramita Palit, Himabindu Kudapa, Robert Zougmore, Jana Kholova, Anthony Whitbread, Mamta Sharma, Rajeev K Varshney

**Affiliations:** a Research Program- Genetic Gains, International Crops Research Institute for the Semi-Arid Tropics (ICRISAT), Patancheru, India; b CGIAR Research Program on Climate Change, Agriculture and Food Security program (CCAFS), Bamako, Mali; c Research Program- West & Central Africa, ICRISAT, Bamako, Mali; d Research Program- Innovation System for Drylands, ICRISAT, Patancheru, India; e Research Program- Asia, ICRISAT, Patancheru, India

**Keywords:** Carbon dioxide, climate change, legumes, molecular intervention, physiology

## Abstract

How unprecedented changes in climatic conditions will impact yield and productivity of some crops and their response to existing stresses, abiotic and biotic interactions is a key global concern. Climate change can also alter natural species’ abundance and distribution or favor invasive species, which in turn can modify ecosystem dynamics and the provisioning of ecosystem services. Basic anatomical differences in C_3_ and C_4_ plants lead to their varied responses to climate variations. In plants having a C_3_ pathway of photosynthesis, increased atmospheric carbon dioxide (CO_2_) positively regulates photosynthetic carbon (C) assimilation and depresses photorespiration. Legumes being C_3_ plants, they may be in a favorable position to increase biomass and yield through various strategies. This paper comprehensively presents recent progress made in the physiological and molecular attributes in plants with special emphasis on legumes under elevated CO_2_ conditions in a climate change scenario. A strategic research framework for future action integrating genomics, systems biology, physiology and crop modelling approaches to cope with changing climate is also discussed. Advances in sequencing and phenotyping methodologies make it possible to use vast genetic and genomic resources by deploying high resolution phenotyping coupled with high throughput multi-omics approaches for trait improvement. Integrated crop modelling studies focusing on farming systems design and management, prediction of climate impacts and disease forecasting may also help in planning adaptation. Hence, an integrated research framework combining genomics, plant molecular physiology, crop breeding, systems biology and integrated crop-soil-climate modelling will be very effective to cope with climate change.

## 1 Introduction

Feeding a growing population in the face of a changing climate poses a major challenge since it involves maintaining the genetic gains needed to sustain the productivity of major crops. There has been an unprecendented urgency and greater momentum in recent decades to find global solutions to this challenge. Greenhouse gases have increased since 1750, with CO_2_, methane and nitrous oxide rising by about 40%, 150% and 20%, respectively [1]. Global warming triggered by increased greenhouse gases, especially CO_2_ (carbon dioxide), poses a serious threat to crop productivity across the globe [1]. The Intergovernmental Panel on Climate Change (IPCC) 2018 special report on the “Impact of global warming of 1.5 °C (SR 15) above pre-industrial levels” pledges to limit global warming to 1.5 °C, which requires that “CO_2_ emissions need to fall 45% from 2010 levels by 2030, and reaching ‘net zero’ around 2050” (https://report.ipcc.ch/sr15/pdf/sr15_spm_final.pdf). A recent report curating 174 papers, including 1540 experiments on the effects of ambient temperature, tropospheric CO_2_ and O_3_ concentration, water availability and salinization estimated the mean effect of standardized environmental changes. It revealed that mean yield (95% confidence interval) and reported yield changed in all vegetables and legumes, ranging from a 22% variation for a 250 ppm increase in CO_2_, 8.9% for a 25% increase in O_3_ and 31.5% reduced mean yields with a 4 °C increase in temperature [2].

Legumes, also known as ‘plant meat’, are an excellent source of protein that play an important role in meeting food security goals (https://www.un.org/sustainabledevelopment). The synergistic interplay of existing abiotic and biotic stresses with rising CO_2_ levels, especially in legumes has been revealed with a combination of heat and drought stresses in legumes like common bean (*Phaseolus vulgaris*) and soybean (*Glycine max*) and cereals like sorghum (*Sorghum bicolor*) and barley (*Hordeum vulgare*) [3]. This multifaceted and alarming scenario is being addressed by scientists in various ways – by focusing on individual stressors, or combined stressors like elevated CO_2_ and existing biotic and abiotic stresses through physiological, biochemical and molecular studies. This paper endeavors to address various perceptions and priorities revolving around these issues. It focuses on the effect of elevated CO_2_ (a major greenhouse gas) and explores possible strategies to tackle climate change that might contribute to better genetic gains in legumes.

## 2 Major physiological and biochemical alterations in legumes triggered by elevated CO_2_

Elevated CO_2_ has been reported to stimulate plant growth under nitrogen-sufficient conditions, but under nitrogen-starved conditions, it may have the detrimental effect of reducing plant growth by altering its primary metabolism [4]. The anatomical differences between C_3_ and C_4_ plants and their different ways of sequestering carbon through 3C and 4C compounds, respectively, have drawn the attention of climate scientists. The expected benefit of elevated CO_2_ on C_3_ plants was initially predicted to outdo that of C_4_ plants. However, a recent study by Reich et al. [5] over a 20-year period reported an initial biomass increase in C_3_ grasslands for over a period of eight years, after which the pattern reversed. Similarly, the duration of an experiment (short term or long term exposure) is decisive to the effect of elevated CO_2_ [6].

**Fig. 1:**
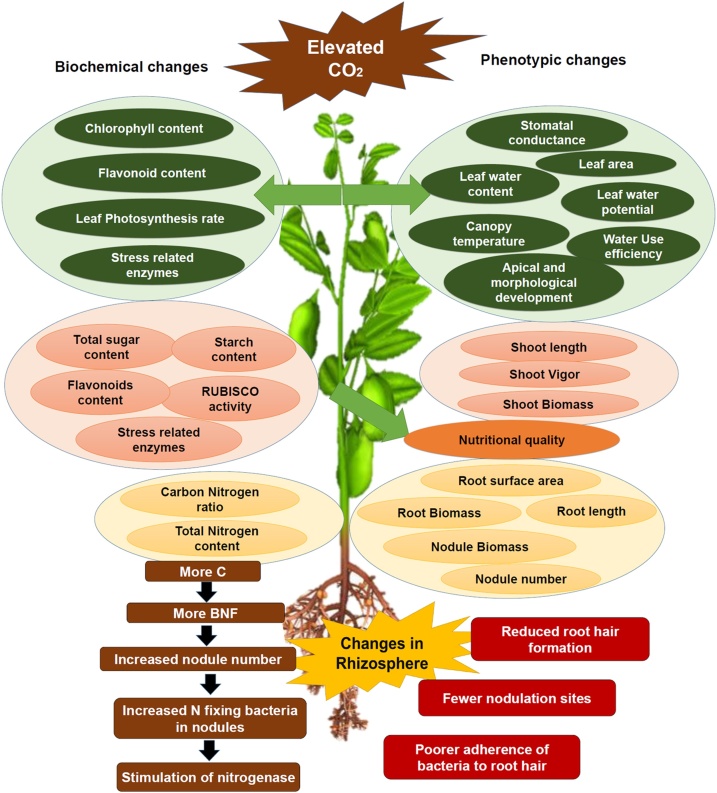
Major physiological traits affected by elevated CO_2_ and elevated temperature. Biochemical and phenotypic changes in legumes when exposed to elevated CO_2_ alone (left) and along with increased temperatures (right). The major phenotypic and biochemical parameters are described in detail in the review section 1. The bottom part of the figure shows the sequential changes in rhizopsphere when exposed to elevated CO_2_ causing increased C which affects Biological Nitrogen Fixation (BNF) with consequences on nodulation in roots.

A range of physiological and biochemical alterations take place in plants exposed to elevated CO_2_. In the case of legumes, elevated CO_2_ also affects the nutritional quality, nodulation, causes changes in rhizosphere, Biological Nitrogen Fixation (BNF), etc. The changes evident in important physiological traits of legumes due to elevated CO_2_ level have been summarized in Fig. 1. Major phenotypic and biochemical parameters of legumes were affected by elevated CO_2_. So are the sequential changes in rhizosphere under excess C (outcome of elevated CO_2_) conditions. The increase/decrease in physiological parameters upon elevated CO_2_ exposure (Table 1**)** are explained in detail in the following sections.

**Table 1:** Physiological trait alterations under elevated CO_2_ conditions along with other stress responses in selected legumes and other key crop species.

Crop	Stress imposed (CO_2_ level and others)	Trait [increased (↑), decreased (↓), not affected (-)]	Reference
Soybean	aCO_2_ (ambient CO_2_) of 390 ppm or eCO_2_ (elevated CO_2_) of 550 ppm	Nodule number per plant (↑), nodule fresh weight per plant (↑), nodule density (↑), single nodule N fixation (↑), seed yield (↑), proportion of remobilized N in seeds (↓) and shoot N concentration (↓)	[29]
	eCO_2_ of 550 ± 30 ppm and aCO_2_ of 390 ± 30 ppm	At mature stage: Protein content (↓), fatty acid content (↑), total free amino acid (↓) total and specific isoflavons (↑), concentrations of potassium (K), calcium (Ca), magnesium (Mg), phosphorous (P) and sulphur (S) (↓), zinc (Zn), iron (Fe) (↓) and Mg, S, and Ca (↑)	[98]
Mung bean	400 or 700 μmol/mol CO_2_+heat + ABA	Above ground biomass (↑), growth indices (↑), nitrogen balance index (NBI) (↑), flavonoids (↑), shoot/root mass ratio (↑) and chlorophyll (↓)	[99]
	eCO_2_ levels of 550 and 700 μL/L	Plant height (↑), leaf area (↑), total dry matter (↑), pod and seed yields (↑)	[53]
Peanut	CO_2_ levels: 400 ppm and 700 ppm	Total biomass (↑) and final seed yield (↓)	[100]
	Temperatures: 33/21 °C (TA), 35.5/23.5 °C and 38/26 °C		
	CO_2_ levels of 375 ppm and 700 ppm + Temp: 28 °C and 32 °C	Transpiration equivalent (↑)	[101]
Field pea	aCO_2_ level of 390 ppm or eCO_2_ of 550 ppm with N treatments by adding Ca(^15^NO_3_)_2_ at either 10 (Low N) or 25 (High N) mg N/kg soil.	Root dry weight (↑), shoot dry weight (↑), root shoot ratio (↓), biomass (↑), soil nitrogen (↓), nodule mass and size (↑) and leghemoglobin content (↓)	[8]
Rice	aCO_2_ of 374-386 μmol/mol or eCO_2_ of 571, 588 and 590 μmol/mol	Grain protein (↓), grain micronutrients (↓), Fe and Zn concentrations (↓) and vitamin content (↓)	[102]
Wheat	CO_2_ above normal levels (365 μmol/mol) and FACE (186 μmol/mol) above ambient + two levels of soil nitrogen supply (350 and 15 kg/ha of nitrogen, NH_4_NO_3_, applied in the irrigation water)	Total activity of ribulose-1,5-bisphosphate carboxylase/ oxygenase (Rubisco) (↑), leaf content of Rubisco (↑) and Light Harvesting Chlorophyll a/b protein associated with Photosystem II (LHC II) (↑)	[103]
Wheat, ryegrass, clover, potato, grape, rice, barley, sugar beet, soybean, cassava, rapeseed, mustard, coffee (C_3_ crops) and sorghum and maize (C_4_ crops)	aCO_2_ of 353 ppm and eCO_2_ of 550 ppm	Shoot biomass (↑), evapotranspiration (↓), biomass (↑), yield (↑) and canopy temperature (↑)	[3]
Quinoa	aCO_2_ of 400 and eCO_2_ of 600 μmol/mol at 20/14 °C day/night temperature, with or without exposure to day/night temperatures of 35/29 °C (“high”) for seven days during anthesis	Leaf photosynthesis and stomatal conductance (↓), Harvest index (↑) and total dry biomass (↑)	[104]
Chinese yam	aCO_2_ and eCO_2_ (ambient +200 μmol/mol) and two mean air temperatures of 22.2 °C and 25.6 °C	Plant growth and vigor (↑), dry weight (↑) and germination percentage (↑)	[105]
Potato, tomato, lettuce and other vegetables	aCO_2_ of ≥200 and ≤450 μmol/L and eCO_2_ of 540 and 1200 μmol/L	Fructose (↑), glucose (↑), total soluble sugar (↑), total antioxidant capacity (↑), total phenols (↑), total flavonoids, ascorbic acid (↑) and Ca (↑) in the edible part of vegetables, protein (↓), nitrate (↓), Mg (↓), Fe (↓) and Zn (↓), total chlorophyll (-), carotenoids (-), lycopene (-), anthocyanins (-), P (-), K (-), S (-), Cu (-) and Mn (-)	[106]
*Trifolium pretense* (legume) and *Agrostis capillaris* (grass)	aCO_2_ of 400 μmol/mol and 700 μmol/mol and under drought with varying soil water content up to 15%	Leaf water potential (↓), root shoot ratio (↑) and leaf water area (↓)	[41]
*Caragana microphylla* Lam (sub-shrub legume species)	aCO_2_ of 380 μmol/mol and eCO_2_ of 760 μmol/mol + two nitrogen levels (0 and 17.5 g N/m/year)	Net photosynthesis (↑), above ground growth (↑), root biomass (-), root shoot ratio (-), symbiotic nitrogenase activity (-) and leaf N content (-)	[107]

### 2.1 Altered shoot and root length, biomass and plant senescence

Significant increase in shoot and root length (due to enhanced vigor) are major traits that can be attributed as the initial effects of elevated CO_2_ in plants. The effects of elevated CO_2_ on carbon partitioning and photosynthesis with special reference to root sugar metabolism was reviewed by Thompson et al. [7]. A varied response is often seen in different plant species including legumes depending on the site of carbohydrate allocation, whether it is seeds, shoots, leaves or roots [8]. For instance in chickpea (*Cicer arietinum*), a significant increase in plant height i.e., shoot length, but a decrease in chlorophyll content has been reported under elevated CO_2_ [9]. An increase in shoot biomass has been reported in field pea (*Pisum sativum*) (36%) and wheat (*Triticum aestivum*) (55%) under 550 ppm elevated CO_2_ [3]. A meta-analysis of free-air CO_2_ enrichment (FACE) and open top chamber (OTC)-based experiments found a general increase in root biomass, root elongation with branching and increased fine root production in response to elevated CO_2_ [10]. In soybean, elevated CO_2_ (800 ppm) increased biomass, enhanced photosynthesis and reduced stomatal conductance, which depends on adequate nutrient (potassium) supply [11]. Increased shoot and root lengths, biomass and other enhanced growth parameters in mung bean (*Vigna radiata*), peanut (*Arachis hypogaea*), pea, soybean and other plant species are shown in Table 1.

Elevated CO_2_ combined with limited nitrogen (N) promotes the progression of plant senescence, such as leaf yellowing and anthocyanin accumulation in *Arabidopsis* [12]. It can also enhance the senescence rates as observed in flag leaves of rice and wheat [13]. In legumes (chickpea), senescence at higher levels of CO_2_ occurred, following a decrease in chlorophyll content, Nitrogen Balance Index (NBI) and insect-plant interactions. All these traits were attributed to low N content in the leaves [14].

### 2.2 Altered stomatal regulation and its consequences

**Fig. 2:**
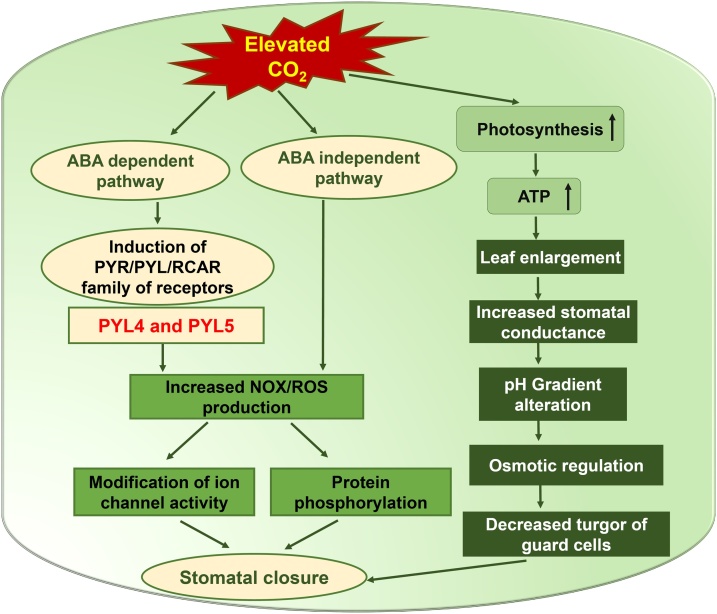
Cumulative effect of elevated CO_2_ and drought on stomatal behavior. Increased CO_2_ modulates OPEN STOMATA1 through ABA-dependent and ABA-independent mechanisms via increased NOX, ROS production and modulation of ion channel activities which in turn changes osmotic regulation, pH, protein phosphorylation and turgor pressure of guard cells. Additionally, increased rate of photosynthesis and ATP production show an additive effect on leaf enlargement and canopy temperature. The molecular mechanisms underlying elevated CO_2_-induced closure and reduction in stomatal density involve generation of reactive oxygen species. The pathway essentially has a bifurcation involving ABA and PYR/RCAR family of ABA receptors through guard cell ABA signaling pathway, acting through a loop-mediated mechanism where CO_2_ induced an increase in ABA, which in turn increases the sensitivity of the system to elevated CO_2_. CO_2_ signal transduction pathway via ABA-OST1/SnRK2.6 shows that basal ABA signaling and OST1/SnRK2.6 are required to facilitate stomatal response to elevated CO_2_. Although ABA and increased CO_2_ induce PYR/PYL/RCAR family of ABA receptors in a stimulus specific manner, in the responses to CO_2_, PYL4 and PYL5 are crucial.

Stomatal regulation of water use efficiency (WUE) i.e., the ratio of photosynthetic and transpiration rates at the leaf level, is a potential trait related to plant productivity that varies with changes in CO_2_ concentration. Decreased stomatal conductance increases WUE and soil water availability [15]. With CO_2_ enrichment under moderate drought conditions, increased leaf area improves water status [16]. However, the larger plant size achieved under elevated CO_2_ can further enhance water use, causing deterioration in plant water status [17]. Increasing CO_2_ concentration in the atmosphere would maintain optimal CO_2_ concentration in the sub-stomatal chamber at the lower level of the stomata opening, resulting in lower rates of transpiration. Therefore, it is expected that the higher CO_2_ conditions brought about by climate change will have a beneficial effect on overall plant water balance and productivity. The regulatory effect of elevated CO_2_ on stomatal development and conductance in tropical forage legume *Stylosanthes capitata* Vogel (C_3_) was reported recently [18]. The detailed mechanism of stomatal behavior upon elevated CO_2_ level along with a crosstalk over drought signaling network was reviewed by Xu et al. [15] and recently updated by Hsu et al. [19]. The molecular mechanism underlying elevated CO_2_-induced closure and reduction in stomatal density has been shown in Fig. 2.

### 2.3 Photosynthetic rates, sugar content, root sugar signaling and plant hormonal network interlinkage triggered

**Fig. 3:**
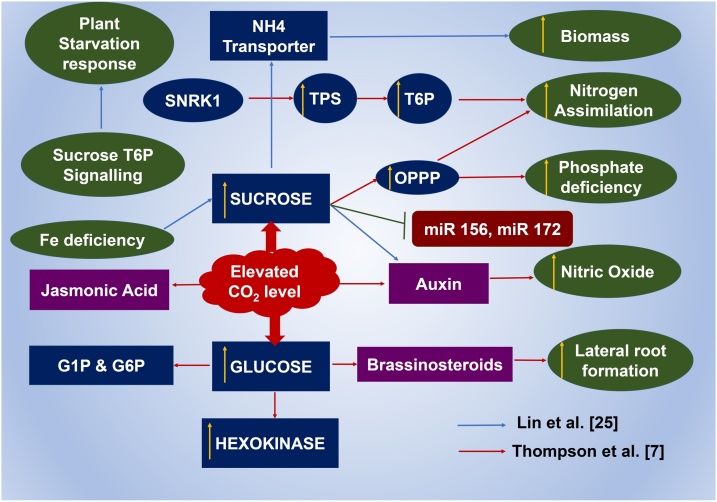
Probable model on elevated CO_2_-mediated response in sugar signaling crosstalk.

Through increased availability of carbon, elevated CO_2_ may augment photosynthesis in plants by shifting the increased sugar levels towards greater sink utilization. This excess sugar (carbohydrate or non-carbohydrate) is stored in various parts of the plant depending on the plant species/cultivar. Several studies have documented the effect of elevated CO_2_ on physiological parameters in legumes, such as increased photosynthetic rates in soybean, dry bean, peanut and cowpea (*Vigna unguiculata*). Carbohydrate accumulation has increased under elevated CO_2_ conditions in soybean [20], dry bean [21] and cowpea [22]. Increased photosynthetic capacity was observed in soybean when grown under 660 μmol/mol of CO_2_ [23]. Increase in starch, reduced sugar content and total non-structural carbohydrate (TNC) content in soybean grown under 800 μmol/mol CO_2_ has also been reported [24]. Increase in CO_2_ often triggers various plant hormone signaling networks, including preferential root growth due to increased shoot biomass and root IAA (Indole Acetic Acid, an Auxin homologue) content and shoot IAA biosynthesis. Sucrose-mediated plant hormone network may be triggered by elevated CO_2_ conditions. For instance, increased sucrose may act through an increase in nitric oxide content (especially in Fe deficient plants) causing FIT- [a basic helix–loop–helix (bHLH) transcription factor] mediated transcriptional regulation, ferric chelate reductase (FRO2) and the ferrous iron transporter (IRT1) genes and induce iron uptake [25]. IAA content in tomato (*Solanum lycopersicum*) roots increased by 26.5% with elevated CO_2_, along with increased ethylene and repression of jasmonic acid synthesis [26,27]. Thus, it is evident that climate change components like elevated CO_2_ have a direct role in existing stress-inducing hormonal networks in plants, hitherto fully unexplored. The crosstalk among different components acting on the sugar signaling network affected under elevated CO_2_ conditions is presented in Fig. 3.

### 2.4 Altered nitrogen balance affecting nutritional quality of legumes

In general, diminished crop nutritional quality reflected in decreased protein concentration in vegetative tissues and grains results in a major economic loss. However, the symbiotic nitrogen fixing capacity of legumes helps in less affecting the carbon-nitrogen balance, provided N_2_ fixation is stimulated along with greater yield [28]. It is also postulated that legumes may alleviate the effect of photosynthetic acclimation under elevated CO_2_ through greater allocation of photosynthates to root symbionts and by maintaining N content by symbiotic nitrogen fixation [8]. In the case of soybean, total protein, flavonoids and free amino acid content significantly decreased at plant maturity stage as a result of elevated CO_2_ but had no influence on the plant’s edible stage. It has been shown that iron (Fe) and zinc (Zn) content decreased, while sulphur (S), phosphorous (P) and calcium (Ca) increased in a stage-specific way. The reported fall in the nutritional quality of soybean might be due to the smaller sample size. This needs to be considered before concluding potentially significant changes in those studies [29]. Dietterich et al. [30] analyzed 18 cultivars each of rice and wheat, 7 of soybean, 5 of field pea and 1 of sorghum under ambient CO_2_ (364–386 ppm) conditions versus elevated CO_2_ (546–586 ppm) conditions through a three-year (1998-2001) period. The study reported decreased nutritional content, especially Zn, Fe and protein concentrations in those crops based on their functional type (C_3_/C_4_ photosynthetic pathways) and cultivar-specific responses. Importantly, it concluded that C_3_ grasses and legumes were consistently affected while C_4_ plants were less affected [30].

### 2.5 Alteration in nodulation and rhizosphere of legumes

Stimulation of nitrogen fixation is often reflected in increased nodule size, nodule number or stimulating nodule activity (amount of N_2_ fixed per unit mass) or all of them [28]. A recent study on root nodulation and plant growth in *Medicago sativa* showed that the positive effect of elevated CO_2_ in growth can be diminished by elevated temperature, whereas silicon supplementation increased the growth under different levels of elevated CO_2_ and temperature [31]. A number of studies have reported that elevated CO_2_ increased nodule number and biomass in chickpea and other legumes. Increased nodule size and number along with plant nitrogen content with enhanced biomass/seed yield has been observed in a number of legume species such as white clover (*Trifolium repens*), lupin (*Lupinus albus*), pea and soybean [8,9,32]. Increased biomass has also been reported in soybean [29] and common bean [28]. A meta-analysis reported about 38% greater N_2_ fixation under elevated CO_2_ because of 33% higher nodule number, 39% higher nodule biomass and 37% higher nitrogenase activity in legumes [33]. A two-year-long FACE-based experiment in lentil (*Lens culinaris*) under ambient and elevated CO_2_ conditions (400 ppm and 550 ppm, respectively) showed higher stimulation in N_2_ fixation. It was expressed through greater nodule number (+27%), mass (+18%) and specific fixation activity (+17%) under well-watered conditions than in the low rainfall/dry season [34]. Hence, it was concluded that benefits of elevated CO_2_ may only be advantageous where other abiotic parameters such as plant water supply were not limiting during grain filling stage [34].

The ability of legumes to fix atmospheric nitrogen through symbiosis with soil bacteria (rhizobia) in nodules is highly sensitive to environmental stresses. Hence, climate change would likely affect symbiotic fixation either directly by impairing rhizobia survival, rhizobia competitiveness, nodule formation, growth, or activity, or indirectly by modifying carbon supply to nodules [35]. This may also happen by penalizing legume dry matter which diminishes with a proportional dependence on nitrogen fixation [36].

The elevated CO_2^-^_mediated stimulation of BNF in legumes is strongest upon immediate exposure to it [33], but under nutrient abundant conditions [28]. The possible mechanisms are through an increase in N_2^-^_fixing bacteria in rhizosphere, increased number of nodules nesting N_2^-^_fixing bacteria and enhanced nitrogenase activity [37]. An increase in carbon allocation towards the root was reported to promote rhizospheric activity of BNF [31,37]. The variability of rhizobia along with root morphological changes have been shown to enhance plant nutrient absorption [33,38]. Also, under elevated CO_2_ conditions, cyanobacterial inoculation and higher P doses have led to enhanced root growth and N_2_ fixation and availability of soil nitrogen [39]. Root nodule symbiosis is temperature dependent; for legumes the optimum temperature for this to occur is around 15-25 °C. Hence, as predicted, a mere rise of 2 °C would take a toll on the development and functionality of root nodulation. It would accelerate nodule senescence through plant-mediated mechanisms like reduced root hair formation, fewer nodulation sites and scarcer adherence of bacteria to root hairs [37].

## 3 Impact of elevated CO_2_ interaction with other abiotic stresses

The impact of elevated CO_2_ on a plant is dependent on other environmental factors such as water deficit stress, temperature, etc. For instance, it has been reported [3] that elevated CO_2_ induced a 10% decrease in evaporation rate in both C_3_ and C_4_ plants. This caused a 0.7 °C elevation in canopy temperature coupled with a 19% yield increase in C_3_ crops. There is evidence that an increase in CO_2_ has also phased down the effect of oxidative stress [40]. A recent comparative study on drought, elevated temperature and elevated CO_2_ effects in grasses and legumes revealed drought-induced inhibition of plant growth, photosynthesis and stomatal conductance. In this case, elevated CO_2_ negatively impacted osmolytes and antioxidants. Additionally, oxidative stress parameters were more reduced in legumes, whereas photosynthesis and chlorophyll levels were more protected in grasses. The study concluded that impacts of elevated CO_2_-mediated mitigation of drought stress is stronger in legumes than in grass species [40]. In this section, the interaction of elevated CO_2_ with water deficit stress and altered temperature has been discussed in detail. Opinions vary on how elevated CO_2_ affects water relations and associated drought tolerance mechanisms. This is so because it is the soil water status that mostly determines whether elevated CO_2_ conditions would be beneficial to the plant’s response. While some studies have reported reduced transpiration under elevated CO_2_ conditions [41], others have reported unaltered transpiration [42] and yet some others have indicated a negative effect [17]. Similarly, osmotic adjustment in drought under elevated CO_2_ conditions is also under debate [8]. Some studies have reported that higher growth rate in the leaf would decrease solute concentration causing minimal osmotic adjustment [43]. Increased drought tolerance due to increased root biomass and fine root production raised the root-shoot ratio under elevated CO_2_ [44]. Elevated CO_2_ may weaken or even prohibit the stimulation of plant growth under water deficit conditions. Thus, crop productivity may decline under predicted future climate conditions in many arid and semi-arid regions worldwide. This would be greater under a combination of elevated CO_2_ and severe drought compared to a combination of elevated CO_2_ and well-watered conditions. Similarly, higher temperature or other extreme environmental factors together with elevated CO_2_ are key climate change factors that could affect plant fitness and flowering related events leading to decreased crop productivity [45]. The earliest studies featuring the effects of long exposure season of CO_2_ in tropical legumes under semi-arid conditions were recorded in peanut. Despite being a C_3_ species, it exhibited photosynthetic rates similar to that of a C_4_ crop under ideal conditions [46]. It was reported that the effects of elevated CO_2_ and plant physiological feedback indirectly ameliorated the drought stress impacts in soybean [29]. The partial mitigation of drought by elevated CO_2_ response is species specific. A study between two grassland species (a legume and a grass) revealed that the negative impact of drought on turgor potential may be avoided by elevated CO_2_ through two different mechanisms. These mechanisms were osmotic adjustment and root to shoot ratio in white clover (legume) and higher leaf relative water content caused by hydraulic conductance in common bent (*Agrostis capillaris*) (grass). However, drought impact was not mitigated in both species through higher soil water conservation [41]. In a recent study, legume faba bean (*Vicia faba*) was grown under ambient (400 ppm) and elevated CO_2_ (550 ppm) conditions under well-watered (80% field capacity) and drought (30% field capacity) treatment. Here, decreased carbohydrate and increased amino acid concentrations in nodules denoted a down regulation of nitrogen fixation. Also, lower seed N concentration has been observed under both elevated CO_2_ and drought conditions [47].

Both warm and cool season species of the legume family, chickpea, pea, common bean, peanut, mungbean, cowpea, etc., have shown severe damage under heat stress during reproductive development [48]. The response and adaptation of legumes under heat stress along with potential combating strategies have been reviewed by Sita et al. [48]. Most of the studies on legumes under heat stress have not taken into account the effect of elevated CO_2_. Interestingly, it has been found that elevated CO_2_ promotes heat tolerance in terms of vegetative growth in legumes such as peanut [49] and cereals such as rice [50], wheat [51] and maize [52]. Heat-tolerant lines of mung bean grown under elevated CO_2_ (550 and 700 μl/L) conditions reported improved growth in plant height, leaf area and total dry matter (13.5%, 67.8% and 46.5%, respectively). It also showed improved pod and seed yields (48.7% and 31.7%, respectively) [53]. Among legumes, increased accumulation of soluble leaf carbohydrates (due to increased photosynthesis) and increased activity of sucrose-phosphate synthase (SPS) and adenosine-5′-diphosphoglucose pyrophosphorylase (AGP) were observed in kidney bean under high temperature, when CO_2_ concentration was about double than under ambient conditions [54]. The increased photosynthesis in C3 plants as an effect of elevated CO_2_ stimulation is attributed to changes in electron transport during light reaction stage. Also, the capacity for carbon fixation and assimilation during dark reaction has an important role in this phenomemon [55].

Drought, when corresponding with high temperature, often poses an additive yet negative impact on crops, playing havoc on their phenotypic and physiological parameters [56]. While there are several studies on combined drought and heat responses in various crops, only a few have considered the effect of elevated CO_2_ along with combined stress response. Studies on legumes, where both drought and heat responses were taken in combination, are rare. Maintaining photosynthetic activity, especially when both drought and heat stress act simultaneously, is an important aspect of plant acclimation. As reported in legumes, this combined stress response often disrupts photosynthesis by altering Rubisco activity and PSII damage [57,58,59].

## 4 Elevated CO_2_ and its interaction with biotic stress-altered pathogen aggravation and virulence

One of the most deleterious effects of changing climate is its adverse effect on biotic stress and on the plant ecosystem [60]. Changing climate has affected pest-crop dynamics through frequent outbreaks and changed geographical distribution of pests, posing an economic threat in legumes [61]. For instance, elevated CO_2_ has increased soybean canopy density and leaf area, which in turn aggravated the expression of soybean fungal diseases, namely downy mildew, brown spots and sudden death syndrome [62]. Sometimes, other abiotic stresses like drought could increase fungal virulence as reported in drought tolerant peanut and *Aspergillus* interaction [63]. However, a combined interaction is not always additive. both unique and common responses have been observed [64].

Increased CO_2_ causes greater photosynthate availability, but reduced foliage quality along with an increased concentration of plant defensive compounds after pest infestation. This in turn affects insect feeding and increases disease incidence and predator parasitism interactions [65]. With increased CO_2_, pod borer (*Helicoverpa armigera*) infestation in chickpea plants revealed altered enzymatic activity. It also caused accumulation of secondary metabolites, total phenols, condensed tannins and increased activity of defensive enzymes [peroxidase (POD), polyphenol oxidase (PPO), phenylalanine ammonia lyase (PAL) and tyrosine ammonia lyase (TAL)]. For example, pod borer-infested plants had higher H_2_O_2_ content whereas the amount of oxalic and malic acids were greater at 750 ppm CO_2_ than at 350 ppm CO_2_ [14]. Hamilton et al. [66] reported increased susceptibility of soybean to herbivory under elevated CO_2_ conditions, with increased level of sugar in the leaves acting as a phagostimulant for the Japanese beetle.

## 5 Molecular interventions for crop improvement under elevated CO_2_

**Fig. 4:**
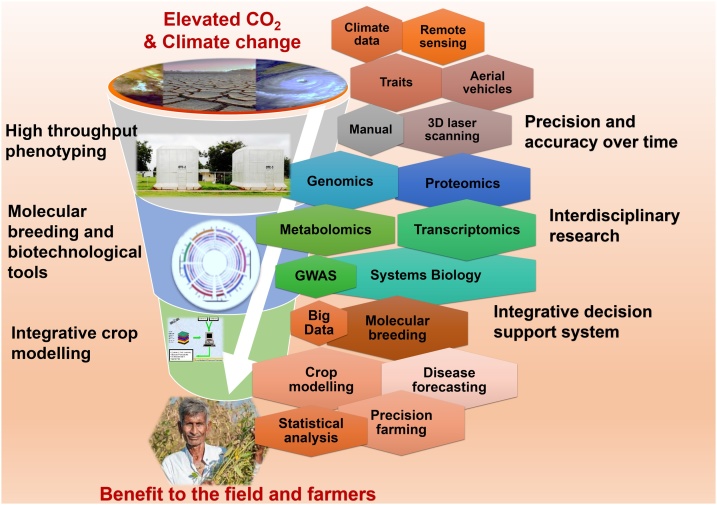
Prospective strategy for climate change research in legumes. A representation of a multifaceted strategy that could be employed to harness cutting edge technologies and greater precision to cope with elevated CO_2,_ and generally with a changing climate.

As mentioned earlier, while elevated CO_2_ may cause greater photosynthate availability, the interaction of elevated CO_2_ with biotic and abiotic stresses calls for the development of climate change-ready crop varieties. In this regard, genomics assisted breeding [67] and other modern approaches can be very powerful tools to develop superior varieties. The last decade saw a surge in genomic resources in legumes, especially in chickpea, pigeonpea and peanut. Varshney [68] summarized the enormous genomic resources i.e. draft genome assemblies, SSR markers, SNPs and genotyping platforms available in these three legumes. These molecular studies can broadly be classified into two categories: one in which genomics studies were undertaken to dissect a physiological trait, followed by a study of its alteration through molecular breeding, transgenic or gene editing approaches. The second group of studies used systems biology approaches integrating transcriptomics, proteomics and metabolomics and deciphered a broader picture of the climate change interaction in plant systems. A majority of molecular studies on elevated CO_2^-^_mediated stress fall in the first group, focused on a particular physiological trait and a study of the changing crosstalk under elevated CO_2_. For instance, the molecular mechanisms underlying elevated CO_2^-^_induced closure and reduction in stomatal density involving generation of reactive oxygen species have been presented in Fig. 4. The pathway essentially had a bifurcation involving Abscisic acid (ABA) and Pyrabactin Resistance/ Regulatory Components of ABA Receptors (PYR/RCAR) family through guard cell ABA signaling pathway. This is a loop- mediated mechanism in which CO_2_ induces an increase in ABA, which in turn increases the sensitivity of the system to elevated CO_2_ [15,69]. Recently, a newer model on CO_2_ signal transduction pathway via ABA-OST1/SnRK2.6 has been elucidated. This model, as indicated in Fig. 4, confirmed that basal ABA signaling and OST1/SnRK2.6 are required to facilitate stomatal response to elevated CO_2_ [19].

Genome wide association studies (GWAS) have been undertaken in several legume crops to address climate resilient traits for crop improvement. For instance in pigeonpea, 292 accessions were used to identify the trait-gene association for 100 seed weight, days to 50% flowering and plant height [70]. Similarly in chickpea, Varshney et al. [71] identified genes associated with drought and heat tolerance traits by sequencing 429 lines and phenotyping 20 lines from one to six locations and seasons. In peanut, marker trait association studies were effectively employed for economic traits like yield component, oil component, drought and disease tolerance [72]; whereas, data of 158 peanut accessions were used for seed and pod traits and domestication of peanut [73]. Superior climate resilient lines or those with improved traits have been developed in legumes [74], such as chickpeas with enhanced drought tolerance (https://www.icrisat.org/first-ever-high-yielding-chickpea-variety-developed-using-marker-assisted-backcrossing-mabc-released-in-ethiopia/; https://icar.org.in/content/development-two-superior-chickpea-varieties-genomics-assisted-breeding) and enhanced resistance to *Aschochyta blight* and fusarium wilt [75]. Similarly, in the case of peanut, leaf rust-resistant [76] and improved oil quality lines have been developed [77]. To comply with the pressing need of addressing the effect of multivariate environmental interactions on climate affecting traits, different prediction models have been applied for superior prediction accuracy in several crops. For example, 13 different prediction models were successfully deployed in chickpea to estimate genotype x environment interaction. This involved coupling phenotyping data of 320 chickpea breeding lines for eight agronomically important traits during three seasons for two locations with genotyping data of DArTseq (1.6 K SNPs) and genotyping-by-sequencing (GBS; 89 K SNPs) [78]. These approaches are important given the changing climate. A number of studies carried out in legumes, Arabidopsis, Jatropha and Bermuda grass for yield and nutritional traits have explored transcriptomic and metabolic changes underlying different physiological parameters including nodulation in legumes upon elevated CO_2_ exposure, combined with or without other abiotic stress. Some of these important studies have been summarized in Table 2.

**Table 2:** Examples of molecular studies in model plants and crops under elevated/low CO_2_ conditions along with other stress responses.

Stress condition	Crop	Molecular tool used	Findings	References
eCO_2_ along with Mg or elevated O_3_	*Arabidopsis*	Transcriptome/ Small RNA-Seq	1) Altered gene expression of the genes involved in regulating flowering time 2) Delayed flowering at eCO_2_ is associated with sustained expression of the floral repressor gene, FLOWERING LOCUS C (FLC), in an eCO_2_‐adapted genotype. 3) Carbon accumulation, defense mechanism redox control, transport, signaling, transport and chromatin remodeling. 4) Alter microRNA expression in *Arabidopsis* growth and development and miR156/157 and miR172 regulated transcriptional network for early flowering. 5) eCO_2_ decreased the expression of genes related to cell redox homeostasis, cadmium response and lipid localization, but enhanced signal transduction, protein phosphorylation, NBS-LRR disease resistance proteins and subsequently programmed cell death (FADB, ATFAH2, WAX2, FATTY ACID DESATURASE 2, FATTY ACID DESATURASE 7, CYTIDINEDIPHOSPHATE DIACYLGLYCEROL SYNTHASE 5 and QUIRKY) in low-Mg shoots. 6) eCO_2_ enhanced the response of lipid localization (mainly LTP transfer protein/protease inhibitor), endomembrane system, heme binding and cell wall modification in high-Mg roots.	[108] [109] [110] [111]
aCO_2_ of 400 μmol/mol and eCO_2_ of 700 μmol/mol concentrations + pea aphid interaction	*Medicago*	Plant iTRAQ proteomic analysis + gene silencing (VIGS)	Susceptible plants: eCO_2_ (↑) PTI defenses including the MAPK signaling pathway (↑), Ca2+signaling pathways (↑), SA signaling pathway (↑) and JA signaling pathway (↓) Resistant plants: silencing of HSP90 in Jester plants impaired ETI signaling and the JA signaling pathway (↓) and nullified the plant‐mediated negative effects of eCO_2_ on aphid performance	[112]
eCO_2_ of 370 μmol/mol and eCO_2_ of 550 μmol/mol and ozone and Japanese beetles	Soybean	Microarray	Leaf-specific transcripts were greater, comprising of Jasmonic acid defense regulatory mechanism (↑), isoprenoids and flavonoids metabolism (↑) related pathways under eCO_2_, elevated O_3_ and eCO_2_ + elevated O_3_ than in aCO_2_, mimicking the scenario of altered atmospheric component in changing climate	[113]
Low CO_2_ treatment, the stomata were first stabilized in 400 ppm CO_2_ balanced opening buffer for 15 min and then exposed continuously to 0 ppm CO_2_ balanced opening buffer for a period of 60 min	Rapeseed	Metabolic profiling	A total of 411 metabolites and 1397 proteins of various pathways are activated at low CO_2_ affecting guard cell stomatal closure and stomatal opening under high CO_2_. Diversion of JA biosynthesis to traumatic acid biosynthesis, the role of melatonin and phytohormone crosstalk, redox regulation and the functions of fatty acid metabolism and Ras-related proteins got affected.	[114]
aCO_2_ of 400 μmol/mol and eCO_2_ of 3000 μmol/mol concentrations	Carrot	qRT-PCR	The transcript profiles of 12 genes related to AsA biosynthesis and recycling were altered in response to eCO_2_ genes, included phosphoglucose isomerase (DcPGI), phosphomannose isomerase (DcPMI), GDP-D-manmose pyrophosphorylase (DcGMP), GDP-D-mannose-3′,5′-epimerase (DcGME), GDP-L-galactose phosphorylase (DcGGP), L-galactose-1-P phosphatase (DcGPP), myo-inositol oxygenase (DcMIOX), ascorbate oxidase (DcAO), ascorbic acid peroxidase (DcAPX), monodehydroascorbate reductase (DcMDHAR), dehydroascorbate reductase (DcDHAR) and glutathione reductase (DcGR). A total of six genes (DcPGI, DcPMI, DcGMP, DcGME, DcGGP and DcGPP) were identified in the L-galactose pathway. DcMIOX were involved in the myo-inositol and D-galacturonic acid pathways, respectively.	[115]
eCO_2_ of 550 μmol/mol in a FACE – 6-year exposure	*Populus*	cDNA Microarray + qRT-PCR	Pathways for secondary metabolism and glycolysis were significantly up-regulated by eCO_2_ during senescence, in particular, those related to anthocyanin biosynthesis. Expressed sequence tags (ESTs) representing the two most significantly up-regulated transcripts in eCO_2_, LDOX (leucoanthocyanidin dioxgenase) and DFR (dihydroflavonol reductase) gave eCO_2_⁄aCO_2_ expression ratios of 39.6 and 19.3, respectively.	[116]
eCO_2_ of 400 μmol/mol, 800 μmol/mol combined with heat stress e.g. at ambient temperature (30/25 °C day/night) and 45/40 °C day/night respectively	Bermuda grass	Metabolic profiling	Important metabolic pathways during which proteins and metabolites were up-regulated, including light reaction and TCA cycle, amino acid metabolism as well as the GABA shunt.	[117]

In addition to genomics-assisted breeding approaches, the post-genomic era has seen transgenic research shift to a much speedy orientation through the inclusion of genome editing technologies like Zinc Finger Nucleases (ZFNs) [79], Transcription Activator Like Effector Nucleases (TALENs) [80] and Clustered Regularly Interspaced Short Palindromic Repeats/CRISPR associated 9 (CRISPR/Cas9) [81]. Various applications of gene editing technology, especially of CRISPR-Cas9, in symbiotic nitrogen fixation (SNF) of legumes have been undertaken by Wang et al. [82]. Using gene editing technology by applying reverse genetics tools, the following genes identified from GWAS have been validated: (1) *Medicago* TnT 177 retrotrasnposon mutant collection [83]; (2) hairpin RNA interference 78 knockout constructs and (3) CRISPR/Cas9 site-specific nuclease (SSN). These include 10 genes responsible for natural phenotypic variation in rhizobia-legume symbiosis [84]. These kind of strategies combining multiple approaches are the need of the future for crop improvement under changing climate.

To address the complexity of climate stressors with larger datasets, the integrative systems biology approach precisely uses multi-dimentional networks through mathematical modelling. This approach is at a nascent stage, especially to study climate resilient traits. However, its components like the gene regulatory networks are being used to integrate and analyze complex bio-molecular network systems at structural dynamic levels [85]. This was done in the documentation of a cohort of transcription regulators, where two published microarray datasets of infection genes expressed in nodule and root hair of *Medicago* have been integrated through a single cell systems biology approach [86]. To explore the genetic basis of the restricted scattered occurrence of root nodule symbiosis, the genomes of 10 plant species of legumes of nodule morphotypes were sequenced. A genome-wide comparative analysis of 37 species revealed signatures of multiple independent loss-of-function events in the indispensable symbiotic regulator Nodule Inception (NIN) in 10 out of 13 genomes of non-nodulating species. This led to an interesting view of the role of selection pressure (a climate modulation will be evident) against symbiosis [87]. However, the integrative approach of coupling omics and physiological parameters are limited to transcriptome- and metabolome- based studies on plants under elevated CO_2_ condition. It is yet to pave its way towards large scale systems study.

## 6 An integrated research framework for the future

The discussion and evidence presented clearly illustrate that the effect of elevated CO_2_ under a changing climate scenario is multifaceted and aggravated by the overlapping interaction of stressors. The notion that CO_2_ has beneficial effects in terms of increased productivity is now being questioned since the photosynthetic fertilization effect is short term and often not time-tested for major crop species. The IPCC 2018 special report highlights a number of policy level approaches that are aimed at limiting greenhouse gas emission. It is important for the scientific community to be prepared with suitable research outcomes to cope with the effects of elevated atmospheric CO_2_ levels. In this regard, an integrated framework combining different biological disciplines is very much required (Fig. 4).

While significant advances have been made in crop genomics, systems biology and genomics-assisted breeding, the success of trait dissection and trait deployment is very much dependent on the quality and precision of phenotyping. Recent advances in plant phenotyping using high throughput phenotyping tools have revolutionized the uptake of phenotype and allelic information in a more precise and robust way and complemented high throughput genomic resources [88]. Variations in field experiments due to environmental factors like elevated CO_2_ can be overcome by using highly flexible, non‐destructive robotic measurement platforms with accurate navigation systems, multivariate sensor modules and the capability of data acquisition from multiple plots [88,89]. High throughput phenotyping has already been initiated in several legumes [90]. Varshney et al. [90] have listed all the state-of-the-art high throughput phenotyping facilities globally that could be effectively deployed in documenting changes in elevated CO_2_ and other climatic factors in legume crops over time [90,91].

Crop models are also key tools that are playing an increasingly important role in assisting agriculture to adapt to climate change. The models aid in extrapolating the complexity of climate change and help to understand its impact on agriculture. The cumulative effect of biotic stress is often aggravated by abiotic stressors under an elevated CO_2_ scenario. It should be addressed though prediction models coupled with adaptation strategies of Integrated Pest Management (IPM). Recent advances in crop and physiological models to study the effect of climate change impacts [92] could be effectively utilized not only for elevated CO_2_ but also in other climate research areas. For example, in the case of chickpea, the modelling approach has been used to quantify region-specific constraints and yield gaps limiting productivity [93]. Modelling innovations can address concerns on sustainable food production, nutrition and natural resource management challenges under a changing climate scenario [94,95].

In short, genomics, transcriptomics, phenomics and metabolomics approaches have enhanced our ability to understand molecular mechanisms underlying important and complex traits. There is a need now to use a systems biology approach to identify not just one or a few genes/ QTLs but to understand plant biology at the system level under a climate change scenario. Similarly, linking studies from genotype to phenotype levels under changing climate requires crop modelling approaches [96]. In our opinion, an integrated research framework that include genomics/ systems biology and phenomics together with suitable crop models would provide the data-driven advisory on optimum GxExM (genotype x environment x management) for current and projected climate. Interdisciplinary approaches are key to graduating from a descriptive level to an improved quantitative and process level understanding of crop productivity. Furthermore, developing an integrated approach inclusive of the recommendations of statutory bodies, policy makers and stakeholders would in the long run help mitigate the deleterious effects of increased CO_2_. For example, climate-smart agricultural initiatives should be modified and modulated through potential feedback from farmers through an integrated decision support system, as has been done in the climate-smart village approach [97]. This way forward will lead to the development of improved crop varieties that can sustain productivity under changing climate.

## Conflict of Interest and Authorship Conformation

All authors have participated in (a) conception and design, or analysis and interpretation of the data; (b) drafting the article or revising it critically for important intellectual content; and (c) approval of the final version.

This manuscript has not been submitted to, nor is under review at, another journal or other publishing venue.

The author(s) declare that they have no conflict of interest.
